# “When a man is stressed, it replicates in the house”: Kenyan women’s perspectives on the influence of male partners on perinatal mental health among women affected by HIV

**DOI:** 10.1371/journal.pgph.0006047

**Published:** 2026-03-04

**Authors:** Hellen Moraa, Joan Mutahi, Winnie Atieno, Grace John-Stewart, John Kinuthia, Manasi Kumar, Mary Marwa, Ben Odhiambo, Felix Abuna, Jillian Pintye, Dalton Wamalwa, Gabrielle O’Malley, Keshet Ronen, Irene Njuguna, Anna Larsen

**Affiliations:** 1 Department of Medical Research and Programs, Kenyatta National Hospital, Nairobi, Kenya; 2 Department of Psychiatry, University of Nairobi, Nairobi, Kenya; 3 Institute for Human Development, Aga Khan University, Nairobi, Kenya; 4 Department of Global Health, University of Washington, Seattle, Washington, United States of America; 5 Department of Pediatrics, School of Medicine, University of Washington, Seattle, Washington, United States of America; 6 Department of Epidemiology, University of Washington, Seattle, Washington, United States of America; 7 Institute for Excellence in Health Equity, New York University School of Medicine, New York, New York, United States of America; 8 Department of Biobehavioral Nursing and Health Informatics, University of Washington, Seattle, Washington, United States of America; 9 Department of Pediatrics and Child Health, University of Nairobi, Nairobi, Kenya; PLOS: Public Library of Science, UNITED STATES OF AMERICA

## Abstract

Mental health conditions are a major public health concern in the African region, where women experience a high prevalence of maternal mental health conditions and limited access to adequate care. This issue is particularly severe among women living with HIV (WLHIV), who face a heightened risk of depression and anxiety. Despite the well-documented association between male partner-related factors and maternal/child health outcomes, their impact on perinatal mental health from the perspective of women is not well understood. We conducted semi-structured interviews and focus group discussions with women affected by HIV. Thematic analysis revealed common sources of mental distress they experienced. Relationship with male partners was identified as an area needing further interrogation to better understand how gender inequities exacerbate maternal mental health outcomes. Male partners emerged as the most significant source of stress for postpartum women, often due to relationship instability, inadequate financial support, and in some cases, verbal or physical abuse. Additionally, women noticed signs of mental health challenges in their male partners, such as anger and chronic stress, and observed that these men often lacked support to address these issues. Other sources of stress included pregnancy-related changes, fear of acquiring HIV among those on HIV PrEP (Pre-Exposure prophylaxis) and fear of transmitting HIV to their babies among those living with HIV. Strategies for coping with perinatal mental health challenges included praying, singing, listening to music, taking walks, social interaction, and participating in church or community-based support groups. Recognizing the significant influence men have on women’s mental health and the interconnectedness of men’s stress and women’s mental well-being, family-oriented mental health programs could be instrumental in improving perinatal mental health.

## Introduction

Globally, mental health conditions during pregnancy and the postpartum period are a public health concern, affecting approximately 10% of pregnant women and 13% of postpartum mothers [1]. Women in the African region are disproportionately affected by perinatal mental health conditions, with an estimated prevalence of depression of 26% in pregnancy and 17% in the first year postpartum [1,2]. Despite this burden, up to 85% do not have access to mental health interventions [3]. In Kenya, studies conducted in urban low-income settings have reported the prevalence of antenatal depression to range between 33% and 38%, and postpartum depression between 19% and 27% [4,5].Perinatal mental health conditions put mother-child pairs at higher risk for a range of adverse health outcomes, including preterm birth, low birthweight, child developmental delay, and maternal death by suicide [6]. Risk factors for developing common mental disorders during the perinatal period range from psychological and biological to social and experiential factors, all of which must be understood in terms of culture and context such as the family and community [7]. Some of the known risk factors for poor perinatal mental health include poor socioeconomic conditions, multiparity, low education, lack of spousal support, intimate partner violence (IPV), stigma, and HIV [8]. A systematic review conducted in Africa reported the prevalence of antenatal depression among HIV-infected women to be 23.4%, and postnatal depression to be 22.5% [9]. Among pregnant women living with HIV (WLHIV), the risk of depression or anxiety is 50% higher [10,11] compared to those not living with HIV, likely due to stress about vertical transmission, HIV-related stigma, and the compounding psychosocial impacts of concurrent HIV-infection and motherhood [7,11]. Kenyan studies report that the prevalence antenatal and postpartum depression among WLHIV ranges between 6–48% [12–14] which aligns with a pooled estimate of 33% reported in systematic review African studies [15].

Culturally, male partners have a strong influence over a family’s financial resources and decision-making, both of which impact the health of women and children [16,17]. Despite the recognized impact on women’s psychosocial and physical health, there is limited research focused on how male partners specifically influence the mental health of perinatal WLHIV or women at high risk for HIV [18]. For example, a recent study in Western Kenya highlighted that a lack of support from male partners negatively affected the mental health of WLHIV, but this issue was not explored in depth [19]. Gaining deeper understanding of how men contribute to women’s poor mental health is essential for improving maternal health outcomes, reducing perinatal distress, and enhancing quality of life for both mothers and their families. Furthermore, understanding the role of partner support could inform the development of more comprehensive and effective mental health interventions for women affected by HIV.

This qualitative study aimed at deepening our understanding of how male partners in settings with high HIV prevalence influence perinatal mental health from the perspective of women. We explored the experiences of women with likely depression or anxiety, including symptoms, coping with mental health challenges, and the role their male partners may play in shaping these experiences.

## Methods

### Study site and population

The present qualitative study recruited participants from two ongoing longitudinal studies involving Kenyan perinatal women affected by HIV to inform client-focused mental health services. The PrEP (Pre-Exposure Prophylaxis) Implementation for Mothers in Antenatal Care extension (PrIMA-X) study enrolled women at risk for HIV in pregnancy or postpartum in 4 maternal child health clinics of Homa Bay and Siaya counties in western Kenya [20]. The HOPE study described elsewhere [21] enrolled women living with and without HIV at 4–10 weeks postpartum in 7 maternal and child health clinics in Nairobi and Western Kenya to compare growth and neurodevelopmental outcomes among HIV-exposed and unexposed infants/children. By nesting our qualitative study within these two longitudinal studies, we were able to include perinatal women both living with and at risk for HIV in our qualitative exploration. This structure also facilitated leveraging existing study infrastructure and rapport between participants and the study team to ensure a foundation of trust.

In the PrIMA-X study, women scoring 10 or higher for likely depression and/ or anxiety on the Center for Epidemiologic Studies Depression Scale (CESD-10) [22] and Generalized Anxiety Disorder Scale (GAD-7) [23] respectively were referred for evaluation and care services. In the HOPE study, women were referred for depression and anxiety evaluation and care services if they scored 10 or higher on the Patient Health Questionnaire (PHQ-9) [24] and 20 or higher on the Kessler psychological distress (K-10) scale [25]. In both parent studies, mental health support was offered to women who screened positive for a mental health condition using routine referral pathways, either to a mental health provider within primary health care facilities or nearby community organizations or lay community persons, they felt most comfortable approaching.

Women were eligible to participate in this qualitative study if they had screened positive for anxiety and/or depression in one of the parent studies. We purposively sampled women for in-depth interviews to achieve an even distribution by location (Nairobi vs. Western Kenya) and to include a mixture of experiences, including those who attended and those who did not, the offered mental health referral. A quarter of in-depth interviews were purposively sampled to be women living with HIV. For FGDs, two groups were conducted in Nairobi, one in Western Kenya; one group in Nairobi was comprised of women living with HIV. Data collection continued until theoretical saturation was reached, meaning no new codes, themes, or insights emerged from the IDIs or FGDs.

### Data collection

We conducted in-depth interviews (IDIs) and focus group discussions (FGDs) with eligible participants using semi-structured guides. The FGD and IDI guides were collaboratively developed and reviewed by the study investigators to ensure the questions were clear, relevant, and aligned with the study objectives. These guides were developed based on the Theoretical Framework of Acceptability [26]. This framework posits that acceptability of healthcare interventions depends on burden imposed by the intervention, affective attitude toward the intervention, ethical consequences of engaging with the intervention, opportunity costs, user experience, and intention or willingness to participate in the intervention. We incorporated these concepts into interview guides. The IDI guide was designed to elucidate women’s descriptions of mental health symptoms, identify sources of mental distress, explore coping strategies, and experiences with referral for mental health services. The focus groups met twice each and used iterative, human-centered design methods to elicit discussion (e.g., journey mapping, role-playing, hypothetical case discussions). The first session focused on women’s strategies for managing mental distress, their preferences for mental health support characteristics, and suggestions for ideal mental health support services. The second session centered on a prioritization exercise of acceptable elements of hypothetical future perinatal mental health services. This study specifically examined the influence of male partners on perinatal mental health among Kenyan women affected by HIV by analyzing data from FGDs and IDIs.

Interviews were conducted from February to March 2023, and FGDs from July to August 2023. The insights obtained from the analysis of IDIs were used to further refine the FGD guide. The IDIs and FGDs were facilitated by two female qualitative researchers with expertise in working with Kenyan perinatal women affected by HIV. Interviews lasted 30–60 minutes, while FGDs were 100–150 minutes and were conducted in quiet and private spaces within the clinic or at nearby community centers. Both IDIs and FGDs were conducted in the participants’ preferred language, which included English, Swahili, or Dholuo, and were audio recorded. At the end of each IDI or FGD, women were offered a debriefing session. If participants were assessed as needing psychosocial support, they were referred for psychosocial support. During the FGDs, a study team member documented significant non-verbal nuances observed from the participants. For the IDIs, the interviewers took detailed notes to record non-verbal cues. Audio recordings from the IDIs and FGDs were transcribed verbatim and translated into English.

### Data analysis

The team adapted Braun and Clarke’s (2013) 15-point checklist for good thematic analysis guided the analytic process, ensuring a rigorous and systematic approach [27].

A five-member coding team made up of four Kenyan researchers and one US-based researcher employed both inductive and deductive approaches to develop a codebook; deductive codes were based on the structure and thematic exploration within the interview guide, while inductive codes were developed by the team reviewing four initial transcripts. The team refined the codebook by testing a subset of transcripts and revised codes and their definitions as necessary. Subsequently, a coding pair sequentially coded each transcript using Atlas.ti software, while engaging in an ongoing review process during which a third member of the team resolved discrepancies. The coding team remained reflexive throughout the process, carefully considering personal biases when interpreting the data. Codes were then organized and examined to search for themes.

### Rigor and trustworthiness

The first author, a Kenyan nurse scientist with extensive experience in the local healthcare system, engaged in reflexive journaling throughout data analysis and manuscript preparation to critically examine her positionality and its potential influence on interpretation. The analysis team included four Kenyan researchers and one U.S.-based social scientist with qualitative research expertise.

To ensure trustworthiness, the team collaboratively developed a codebook and conducted consensus coding on initial transcripts. Subsequent transcripts were independently coded by a pair, with discrepancies resolved by a third coder and team discussion. Regular team meetings with study investigators facilitated oversight of the coding process and refinement of themes.

### Ethics statement

This study was reviewed and approved by the Institutional Review Board (IRB) at the University of Washington, approval number (STUDY00015293) and the Kenyatta National Hospital/University of Nairobi (KNH/UON) Ethics and Research Committee (ERC), approval number P395/05/2022.

### Informed consent

All participants provided written informed consent prior to study participation.

## Results

### Participant characteristics

We conducted 21 IDIs and 6 FGDs (two sessions with three groups of women) with 28 participants (9–10 in each group) among perinatal women affected by HIV. Five of the participants who took part in the IDIs also participated in the FGDs. Of the 44 participants, 15 were purposively sampled to be WLHIV. Among IDI participants, the median age was 30 years (range: 22–44), half were 22–24 months postpartum at the time of the interview, and 90% of respondents had attended their perinatal mental health referral (Table 1).

**Table 1:** Participant Characteristics.

	FGD (n = 28)		IDI (n = 21)	
Demographic characteristics	n	n (%) or Median (IQR)	n	n (%) or Median (IQR)
Age (years)	28	30 (27, 34)	21	30 (22, 44)
Living with HIV	28	9 (32%)	21	6 (24%)
Late postpartum (22–24 months)	28	19 (68%)	21	11 (52%)
Attended mental health referral	28	25 (90%)	21	19 (90%)
Education (years)	26	11 (8, 14)	20	9 (8, 12)
Regularly employed	26	3 (12%)	20	3 (15%)
Married	26	20 (77%)	20	12 (60%)

### Drivers and symptoms of mental distress

Women described mental distress as presenting through a range of interconnected physical and emotional symptoms, including physical pain, disrupted sleep, diminished appetite, mental fog, and feelings of apathy. Some participants reported a decreased sense of purpose, reduced desire to continue living and in more extreme cases, thoughts of self-harm or suicide.

*“At the time I had a headache, even if I take drugs, it does not stop. I felt my heart is paining it was pumping fast.”* (**IDI participant #20, WNLHIV)**

*“When I was pregnant with this baby, I became sick…... My mother-in-law told her son that he is too young to take care of someone like me and that he should leave me. The man left me while I was sick and helpless and went back home to his mother. I have another six-year-old daughter, I sat and told myself that it would be better to die, I wanted to kill my daughter and myself….”* **(FGD 1, participant #1, WLHIV)**

Some women attributed their mental distress as resulting from an interplay of physiological changes during pregnancy and various external social stressors. They noted that the combination of biological shifts such as hormonal changes and physical discomfort, were compounded by stressors in their relational, economic and community environments. This interaction of factors creates a complex source of psychological distress contributing to increased distress and vulnerability.

*“When pregnant we usually go through a lot of challenges … When I was pregnant with my second child, I was going through so much and I was going crazy, I don’t want to overshare.”* **(FGD 2, participant #5, WNLHIV)**

**“***When pregnant, you can feel very stressed, and when you deliver, it can get quite overwhelming … the mood swings, stress at home, heightens during pregnancy.* **(IDI participant #5, WNLHIV)**

Fear related to HIV emerged as a major driver of psychological distress among both groups of women. at-risk women, and the possibility of HIV transmission to their children among WLHIV were major sources of stress. Among women at risk for HIV, their anxiety stemmed from the potential risk of acquiring the virus, whereas women living with HIV described deep emotional distress upon receiving a HIV diagnosis during pregnancy. For women who were already aware of their HIV status by the time they became pregnant, concern over the likely progression of HIV to AIDS-defining illness contributed to additional stress**.** These fears, ranging from their own HIV risk to the potential for vertical transmission, shaped their overall mental health experiences during pregnancy.

“*When I was 3 months pregnant with my son … I found out that I was not only pregnant but I had also been infected with HIV. So, I was very stressed and was not able to share with anyone. At the time I was staying with my grandma, who noted that something was wrong. She advised me to terminate the pregnancy. After learning of my positive status, I was enrolled with the NGO-Dream Girl. I felt sorry for myself, I was confused, depressed, and traumatized. … My parents were also pressuring me to terminate the pregnancy.”* **(IDI participant #7, WNLHIV)**

*“I was very stressed; life was not going to be the same again [following the HIV diagnosis]. I was stressed wondering how I would go through that state, and whether it would affect my children, it really bothered me. At times I would sit, and my mind would drift off”* **(IDI participant #6, WLHIV)**

### Male partners were frequently identified as contributing to mental distress

**Fig 1:**
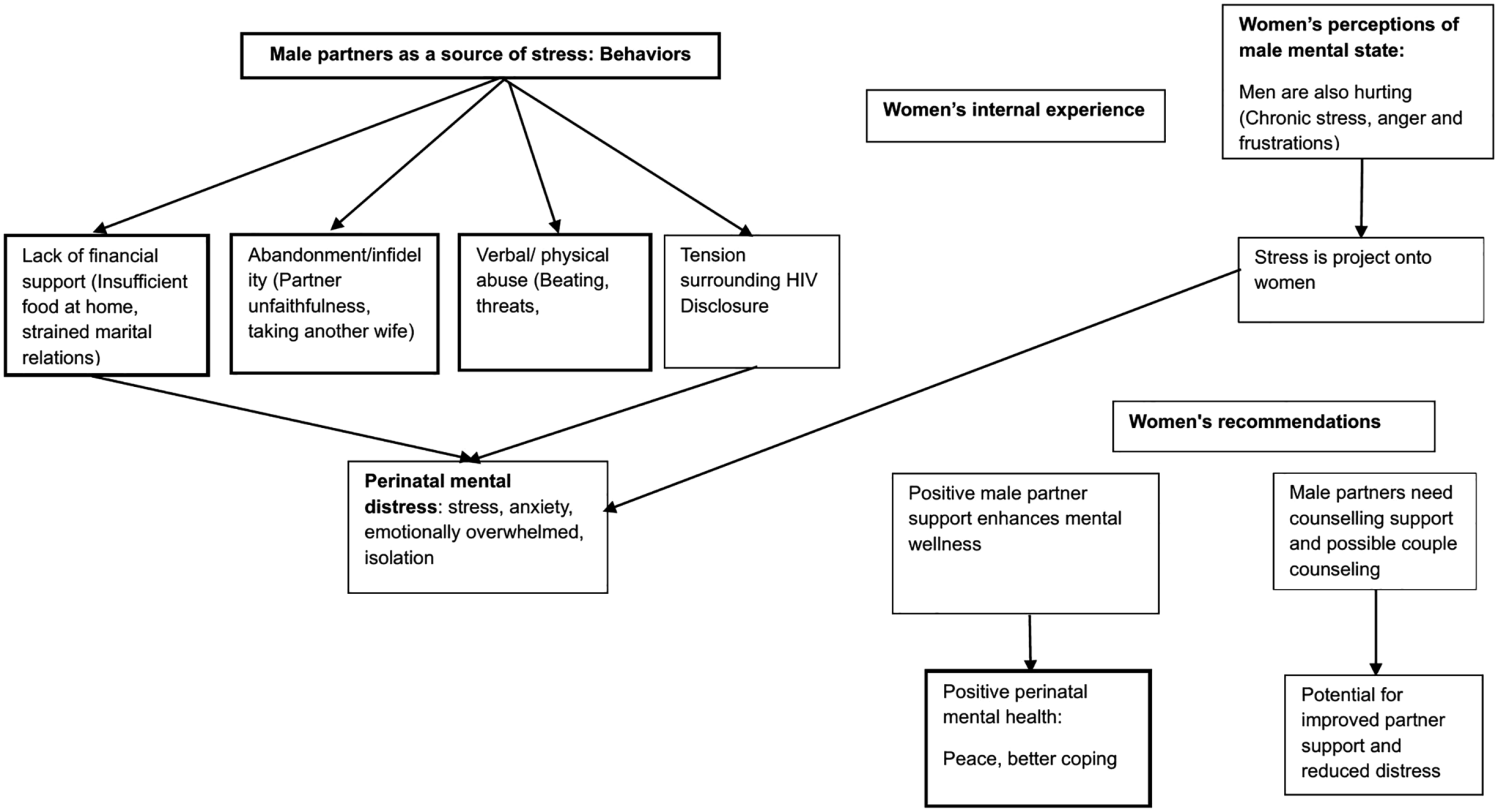
Conceptual framework illustrating pathways linking male partners’ influence on women’s perinatal mental distress. The figure depicts how male partner behaviors contribute to diverse stressors that impact women’s mental health experiences.

Women consistently identified their male partners as a significant contributor to psychological distress during pregnancy and the postpartum period. This is a period, when they were especially vulnerable and their need for support made partner-related stress impactful. Men contributed to this stress through multiple pathways (Fig 1).

### Lack of financial support

During pregnancy and early postpartum period women described their mental distress as related to economic dependency. Whenever male partners did not provide financially, women had to step up and shoulder the financial responsibilities, which heightened feelings of vulnerability and anxiety. Participants also linked poor treatment from partners to their lack of income, describing situations where economic dependence translated to diminished respect, harsher treatment and increased emotional strain. These accounts suggest that financial insecurity and unequal power dynamics within relationships were drivers of stress during the perinatal period.

*“Maybe at home you have stress from your partner, and you don’t want anybody to know about it. My husband doesn’t leave money for food mostly, he sometimes leaves 50 shillings which is not enough to cater for the 3 children and myself.* **(IDI participant #4, WNLHIV)**

### Abandonment

Women’s accounts of partner abandonment and infidelity were interpreted as both emotional and material. They shared how such actions affected their mental health, leading to feelings of betrayal, isolation, and an increased psychological burden. They further noted that after their male partners left, in most instances, they also withdrew financial support, leaving the women as sole providers for their families during a particularly vulnerable pregnancy/ postpartum period.

**“** *Women go through a lot. My personal experience was that I got my babies one after the other…. Their father left and I remained with the children. I got so stressed, and I had to go fend for the children when the baby was still young* **” (FGD 1, participant #2 WLHIV)**

*“They asked me what brings me stress. By that time, my husband had added another wife, so he was stressing me. He was just drinking, and I was feeling like he was behaving that way because I am pregnant. Those are the thoughts that I had”* **(IDI participant #14, WNLHIV)**

### Verbal or physical abuse

Several women reported experiencing physical violence, threats, and verbal abuse from their partners. This intimate partner violence caused profound psychological distress. This caused women constant fear, anxiety, emotional exhaustion, and it even had an impact on their physical well-being. For some, the severity of the abuse compelled them to seek emotional support or relocate from their homes underscoring the extent to which the violence disrupted their sense of safety and well-being.

“*When I was pregnant my husband used to beat and torture me, I completely lost weight. He would sell things in the house to buy drugs. By the time I was almost giving birth, he had sold the blanket. I got high blood pressure when I was almost delivering … I separated from my husband and now live with my three children*.” **(FGD 1, participant #2 WLHIV)**

*“I just left, because he was demeaning me. He beats me and even threatens me with a machete”* **(IDI participant #20, WNLHIV)**

### Tension related to HIV disclosure

Among both women living with HIV and those at risk for HIV, the experiences surrounding HIV disclosure often produced anxiety and compromised relationship stability. Women reported the inadvertent discovery of their male partner’s HIV status which prompted them to seek HIV testing and HIV prevention therapy (PrEP). Others reported finding out about their own HIV status in the context of a partner’s undisclosed or unknown status adding to their emotional burden.

“ [my husband said] ‘*you have been sickly ever since you conceived, have you gone to the hospital?’ and I told him, ‘No I have never gone.’ ‘Why haven’t you gone? Do you want to die? I am tired of burying women!’ and I said, ‘Why?’ He said, ‘You know I am HIV-positive, and I have taken ARVs for twenty years*.’ **(FGD 3, participant #6, WNLHIV)**

“*I did not know that I was positive until I got pregnant with this one and went to the clinic at three months. …. I didn’t know how to break the news to my husband. They told me not to tell him that I was positive but to leave them his number and they would call him and see how to convince him to come and get tested. They called him but he refused to go. So, we started having issues in the house and separated at three months of pregnancy*. (**FGD 1, participant #3, WLHIV**)

### Women acknowledged men are hurting too

Women also perceived that their male partners were also experiencing mental health challenges, often attributed to chronic stress, anger, and personal frustrations. They described how these pressures were sometimes displaced onto them, contributing to relational strain and emotional distress. While women expressed a strong desire for their own mental health support, they emphasized that interventions must address men’s mental health. Without support for male partners, women feared that ongoing partner distress would continue to negatively affect their own mental health.

*“… men are different. There are those who drink and those who stay sober, who do not even go clubbing. Now you can find that yours does not drink but when he leaves in the morning for work, upon coming back just at the doorstep, anything that comes his way he beats up. Meaning that he has stress and cannot tell anyone.”* **(FGD 3 participant #7, WNLHIV)**

**“** *When a man is stressed, it replicates in the house. You will find out that he knocks down everything, beats the children and the wife, quarrels with the wife. Everything that is happening in the house is not as he wants and that there is nothing good you are doing to him, that is when he has a problem* **.” (FGD 3 participant #2, WNLHIV)**

“*But the challenge may be the men, how will they be brought [on] board, so that they may be at a position that we learn together? You see how I wish that the services that I was being given, the counselor … we could be together in the talk, at least somebody to share their minds and the feelings when people are together rather than one. Okay, I’m relieved yes, but back home there, maybe you’re still experiencing the same. At times it’s very hard to cope with the situation.”* **(IDI participant #18, WNLHIV)**

### Women believe male partner support positively impacts their mental health

Participants highlighted that male partner support during the perinatal period can positively impact mental wellness. Although most of them did not have personal experiences of supportive partnerships, they drew on observations from their communities, noting that women with engaged and caring partners appeared happier, more stable and able to cope with stressors. These suggests that male partner support could function as an important protective factor against perinatal mental distress.

“… *But I think that, she has peace in the house, whatever she wants she gets, that man has even her peace at heart, she does not have thoughts of what is going to happen today or in what mood is so and so going to come back in … Now it makes her not have stress. And then also, she is pregnant, and she knows very well that she will get everything the baby wants at whichever time she needs them and in which quantity. Her child will get everything be it food, she will get. ….. Now this makes that woman to be happy and peaceful all the time***.” (FGD 3, participant #5, WNLHIV)**

One participant shared her own positive experience receiving support from her male partner.

*“When I was pregnant with this baby, I was so stressed and I was contemplating whether to abort or commit suicide because my first born is five years old, almost turning six, this second one came, and after two months, I became pregnant with this third one and I didn’t know. When I found out I used to lock myself in the house with the children and when it would become too much, I would pick this one (referring to the second one) and put him on my back and go for a long walk and talk to people on the way and laugh … my husband was also very understanding and he would encourage me and tell me not to think of committing suicide* **” (FGD 2, participant #10, WNLHIV)**

Women gave suggestions that the men should be included in women’s counseling sessions (couple counseling). They were also afraid of continued stress from men who had not received the kind of support the women were receiving. This highlights that HIV care for couples is often delivered individually, underscoring the potential to incorporate couple counseling into the treatment process.

***“****I’m really wondering how they could be brought on board so that all of us, the couples, may have a single talk. This one shares…pours the heart, the other one also pours the heart at least they are being counseled together. And if that one can be done, I think things can really mean to change.”* **(IDI participant #19, WLHIV)**

### Women’s common mental health coping strategies

In the context of limited social support, women adopted various strategies to cope with perinatal mental distress. These self-directed practices included, praying, singing, listening to music, and spending time outdoors served as accessible and meaningful ways to regulate emotions and achieve psychological relief..

“*For me when I feel overwhelmed, I finish with prayers … it helps a lot because sometimes when I am overwhelmed and I pray, I feel relieved immediately after praying. For me it helps better than going to tell someone who is only going to spread it and probably twist the story. So, it is better to just finish with a prayer; I just ask God to give me courage to go through my temptations*.” (**IDI participant #13, WNLHIV**)

*“There are times I wake up and if I feel like I have moods, I will go to the park or go look at cars. Just like that. Or I would go to the flyover and look at cars and relax my mind. Sometimes I would go sit under tree where we live and feel the fresh air and feel relaxed … That at least helps me keep off from thinking a lot*.” (**IDI participant #5, WNLHIV)**

Women also reported that distractions from their mental health concerns were helpful, such as playing with children or socializing either through visits or phone calls to others.

“*I even call some of my friends to the house and tell them to come visit. I would cook for them, I put on some music, and we could dance, and I feel happy, and she would go back to her place*” (**IDI participant #9, WLHIV)**

“*There are times when I see I’m so stressed, I get a ball and my kids, and I go the field to play with the ball. If it’s playing around, jumping I would do so and after I’m happy. The kids at least make me laugh and I turn out to be okay. That helps me to be relaxed and okay*.” **(IDI participant #7, WNLHIV)**

Women living with HIV also noted that once they had come to terms with the HIV diagnosis, it became easier for them to cope. This need could often be bridged through counseling support within the HIV care and treatment programs.

**“** *The struggle was more during pregnancy because after the breakup, it was when I got tested and found out that I was positive. So, I was struggling with accepting myself throughout the pregnancy. Acceptance is the most important thing, once you accept yourself, things become easier* **” (IDI participant #6, WLHIV)**

Women often talked to a pastor or community leader for advice or confided in trustworthy friends and family members. Others also sought counseling services at a health facility. This points to the need to strengthen community structures and healthcare professionals that can offer psychosocial support to women during the perinatal period.

“*With stress, one needs to cool down first. You have to know where you are going, whom you are going to talk to. When I am stressed, I call my mother, and I feel I am ok. But first I ask her where she is and who she is with, if she is free so we can talk. And whenever she hears that, she makes herself available so that we can talk. So, you have to know the timing*.” (**IDI participant #9, WLHIV)**

“*Yes, I had already delivered, and I had a very bad experience caused by the father of my baby. I opted to go for counseling because I thought if I share with a friend, they may misunderstand me and so it was better telling the doctor who wouldn’t*…” (**FGD 1, participant #6, WLHIV**)

Other approaches to coping with mental distress included going to church or women’s groups that were connected to church or HIV care peer support groups.

“*It could even be fellowship members that you’re familiar with and you’re within the church, he/she will support you and tell you what to do as you share what you go through. If you tell them you don’t have food they will tell you they will help with what they have. By the way we have a women’s fellowship in church. We usually tell each other to walk with at least 50 shillings or 100 shillings so that when one of us is down we ask them to say how they really are. If they’re not okay, we ask what they don’t have. If it’s food, we contribute 50 shillings each and she goes home helped*” (**FGD 2, participant #7, WNLHIV**)

## Discussion

Our qualitative study among Kenyan women affected by HIV who had experienced perinatal mental health challenges elucidated important findings about the role of male partners in perinatal mental health. Women reported that mental health challenges often manifested as physical pain, disrupted sleep, lack of appetite, diminished social connections, and unclear thinking. Key sources of stress included the risk of HIV, financial worries, pregnancy concerns or hormonal changes, postpartum-related challenges, and, most prominently, strained relationships with male partners. Women frequently identified male partners as a source of stress during the perinatal period, citing abandonment, infidelity, insufficient financial support, and abuse. Women receiving a recent HIV diagnosis or finding out about their partner’s HIV status faced increased relationship tension. Additionally, women noted that their male partners exhibited signs of mental health challenges themselves, such as anger and chronic stress, and did not have proper avenues for addressing these issues. To cope, women relied on social networks and psychosocial support by attending church, playing with their children, listening to music, going outside, and making social visits or phone calls.

Our findings on stressors during the perinatal period align with another study among women living with HIV in Western Kenya which highlighted relationship stress, financial insecurity, pregnancy and postpartum demands, and lack of support from male partners as leading causes of mental distress [19]. Additionally, a recent systematic review found that inadequate partner support was linked to perinatal distress [28]. Other studies have also found that depression and anxiety symptoms are linked to limited partner support or relationship tension. Our study highlights that male partners play a significant role in contributing to stress among perinatal women experiencing mental health symptoms in high HIV-burden setting. We also highlight the mechanisms through which men cause women distress (Fig 1). This highlights the need for male partner involvement in women’s perinatal mental health. Women in our study recognized that male partners were also facing mental health challenges. They reported noticing the men were stressed and their reactions to these struggles negatively impacted both their relationships and individual well-being of the women. Men have previously been reported to experience mental health challenges during the perinatal period and expressed the need for mental health support [29]. Men’s mental well-being is crucial, as their involvement during the perinatal period has been shown to reduce maternal depressive symptoms in similar contexts [30,31]. Prior studies have also suggested that interventions for couples could be beneficial for supporting mental health during pregnancy and postpartum [32]. Providing this support could reduce the mental distress burden on women, and male partners could potentially be confidants for women facing mental health challenges.

Situating our findings within the Theoretical Framework of Acceptability, male partners currently serve as a reason for non-engagement or discontinuation with perinatal mental health care (“burden”) and contribute to women’s affective attitudes toward available and potential perinatal mental health interventions (“affective attitude”). Women reported willingness to attend available or potential perinatal mental health services (“intention”), yet this intention depended on their male partners’ knowledge of it in some cases, or in other cases women expressed being more willing to engage if mental health services were also provided to their male partners. For some women, male partners’ negative reactions to the woman’s attendance to perinatal mental health services could serve as an unintended negative consequence of the intervention (“ethical consequences”). Overall, male partners played multiple roles in the acceptability of available and potential perinatal mental health services among Kenyan women.

Some strategies that helped women cope with mental distress, such as finding new sources of financial support like paid work or receiving social support from family and friends, are consistent with findings from other studies. These studies have reported that socio-culturally adapted interventions such as the Friendship Bench which utilized lay health workers to offer psychosocial support for women [33,34] and economic empowerment programs [35] can help reduce mental distress among women living with HIV. Unfortunately, WLHIV who are most affected by depression or anxiety may be impaired in seeking work or social support and need interventions to access these strategies [34,36]. Mental health support is also not routinely included for WLHIV and access to intimate partner violence (IPV) care support are limited [12,36]. As a result, many women with potential depression and anxiety are overlooked and do not receive the necessary support for the health and well-being of both mother and child. Previous studies in similar settings have recommended integrating mental health services for WLHIV [12,37].

## Strengths and limitations

To our knowledge, this is the first study in Kenya to explore how male partners influence women’s perinatal mental health. The insights are drawn from the lived experiences of women who screened positive for either anxiety, depression or both during pregnancy or postpartum provide rich, context specific information on an understudied determinant of maternal well-being.

Our study had some limitations. We were not able to interview male partners; all the views regarding male partners were reported by women. The perspective of male partners is important as we seek to understand how to support them in helping their female partners as they face perinatal mental distress. In addition, these interviews and focus group discussions were conducted in service of the parent study goals, thus we did not expressly set out to explore women’s perspectives on male partner influences on their mental health. These themes emerged as inductive findings. Our results were limited to perspectives offered by women and interrogated with follow-up questions from facilitators yet were not directly asked as theoretically guided interview questions. Future studies that include male voices and innately intend to explore the influence of male partners on women’s perinatal mental health would further inform acceptable and effective interventions.

## Conclusion/policy implications

Kenyan women affected by HIV frequently reported experiencing mental health challenges during the perinatal period, with male partners identified as the primary source of stress. Consequently, integrating maternal mental health screening and care into primary healthcare services and promoting the active involvement of male partners could be beneficial. Perinatal mental health services designed for Kenyan women should address prominent sources of stress with interventions tailored for couples, and content on financial empowerment, addressing pregnancy concerns, and support for HIV prevention and disclosure. Interventions aimed at supporting men’s mental health would have a significant impact, not only on their well-being but also on the overall health and stability of families, particularly during the perinatal period. These services could incorporate existing coping strategies used by women, such as involving church or community leaders, forming peer support groups, and including mental health services for men to improve family dynamics.

## Supporting information

S1 Table Additional participant quotations depicting key themes and subthemes. (DOCX)

S1 File Checklist. (DOCX)

## References

[1] Dadi AF, Wolde HF, Baraki AG, Akalu TY. Epidemiology of antenatal depression in Africa: a systematic review and meta-analysis. BMC Pregnancy Childbirth. 2020;20(1):251. doi: 10.1186/s12884-020-02929-5 32345263 PMC7189721

[2] Dadi AF, Akalu TY, Baraki AG, Wolde HF. Epidemiology of postnatal depression and its associated factors in Africa: A systematic review and meta-analysis. PLoS ONE. 2020;15(4):e0231940. 10.1371/journal.pone.0231940 PMC7188237 32343736

[3] Woody CA, Ferrari AJ, Siskind DJ, Whiteford HA, Harris MG. A systematic review and meta-regression of the prevalence and incidence of perinatal depression. J Affect Disord. 2017;219:86–92. doi: 10.1016/j.jad.2017.05.003 28531848

[4] Madeghe BA, Kogi-Makau W, Ngala S, Kumar M. Risk factors and experiences of prepartum depression in urban- low-income settlement Nairobi Kenya: a mixed-method study. F1000Res. 2021;9:1495. 10.12688/f1000research.27434.1 PMC8207804 34211703

[5] McNab SE, Dryer SL, Fitzgerald L, Gomez P, Bhatti AM, Kenyi E, et al. The silent burden: a landscape analysis of common perinatal mental disorders in low- and middle-income countries. BMC Pregnancy Childbirth. 2022;22(1):342. doi: 10.1186/s12884-022-04589-z 35443652 PMC9019797

[6] Dadi AF, Akalu TY, Wolde HF, Baraki AG. Effect of perinatal depression on birth and infant health outcomes: a systematic review and meta-analysis of observational studies from Africa. Arch Public Health. 2022;80(1):34. doi: 10.1186/s13690-022-00792-8 35057865 PMC8772173

[7] Ladur AN, van Teijlingen E, Hundley V. Male involvement in promotion of safe motherhood in low- and middle-income countries: A scoping review. Midwifery. 2021;103:103089. doi: 10.1016/j.midw.2021.103089 34293604

[8] Atif N, Lovell K, Rahman A. Maternal mental health: The missing “m” in the global maternal and child health agenda. Semin Perinatol. 2015;39(5):345–52. doi: 10.1053/j.semperi.2015.06.007 26164538

[9] Abajobir A, Sidze EM, Wainaina C, Gerbaba MJ, Wekesah FM. The epidemiology of maternal mental health in Africa: a systematic review. Arch Womens Ment Health. 2025;28(5):997–1089. doi: 10.1007/s00737-025-01563-4 40220206 PMC12436556

[10] Sowa NA, Cholera R, Pence BW, Gaynes BN. Perinatal Depression in HIV-Infected African Women: A Systematic Review. J Clin Psychiatry. 2015;76(10):1385–96. 26528645 10.4088/JCP.14r09186

[11] Zhu Q-Y, Huang D-S, Lv J-D, Guan P, Bai X-H. Prevalence of perinatal depression among HIV-positive women: a systematic review and meta-analysis. BMC Psychiatry. 2019;19(1):330. doi: 10.1186/s12888-019-2321-2 31666033 PMC6822469

[12] Osborn L, Ronen K, Larsen AM, Richardson B, Khasimwa B, Chohan B. Antenatal depressive symptoms in Kenyan women living with HIV: contributions of recent HIV diagnosis, stigma, and partner violence. AIDS Care. 2022;34(1):69–77. 34579601 10.1080/09540121.2021.1981216 PMC8758509

[13] Hegarty T, McGrath CJ, Singa B, Kinuthia J, John-Stewart G, Pintye J. Postpartum depression and prevention of mother-to-child transmission of HIV in Kenya. J Assoc Nurses AIDS Care. 2019;30(6):675–81. 31259846 10.1097/JNC.0000000000000088 PMC6800757

[14] Yator O, Mathai M, Albert T, Kumar M. Burden of HIV-related stigma and post-partum depression: A cross-sectional study of patients attending prevention of mother-to-child transmission clinic at Kenyatta National Hospital in Nairobi. Front Psychiatry. 2021;11. 10.3389/fpsyt.2020.532557 PMC7947326 33716799

[15] Tadesse G, Rtbey G, Tinsae T, Andualem F, Kelebie M, Kibralew G, et al. Depressive symptoms and its determinants among people living with HIV in Africa: systematic review and meta-analysis. BMC Psychiatry. 2025;25(1):325. doi: 10.1186/s12888-025-06766-8 40175939 PMC11967033

[16] Mkandawire E, Bisai C, Dyke E, Dressel A, Kantayeni H, Molosoni B, et al. A qualitative assessment of gender roles in child nutrition in Central Malawi. BMC Public Health. 2022;22(1):1392. doi: 10.1186/s12889-022-13749-x 35858910 PMC9297552

[17] Tuyisenge G, Crooks VA, Berry NS. “He lets me go although he does not go with me.”: Rwandan women’s perceptions of men’s roles in maternal health. Glob Health Res Policy. 2021;6(1):2. doi: 10.1186/s41256-020-00185-w 33431064 PMC7802268

[18] Sanfilippo KRM, McConnell B, Darboe B, Huma HB, Glover V, Stewart L. The experience of maternal mental distress in The Gambia: A qualitative study identifying idioms of distress, perceptions of contributing factors and the supporting role of existing cultural practices. PLOS Glob Public Health. 2023;3(9):e0002329. doi: 10.1371/journal.pgph.0002329 37676895 PMC10484451

[19] Tuthill EL, Maltby AE, Odhiambo BC, Akama E, Pellowski JA, Cohen CR, et al. “I Found Out I was Pregnant, and I Started Feeling Stressed”: A Longitudinal Qualitative Perspective of Mental Health Experiences Among Perinatal Women Living with HIV. AIDS Behav. 2021;25(12):4154–68. doi: 10.1007/s10461-021-03283-z 33997940 PMC8126180

[20] Marwa MM, Kinuthia J, Larsen A, Dettinger JC, Gomez LA, Awino P, et al. COVID-19 vaccine hesitancy among pregnant and postpartum Kenyan women. Int J Gynaecol Obstet. 2023;162(1):147–53. doi: 10.1002/ijgo.14773 37036449 PMC10330087

[21] Njuguna IN, King’e M, Moraa H, Kumar M, Benki-Nugent S, Wagner AD, et al. Cohort profile: longitudinal and population comparison of children who are HIV-exposed uninfected and children who are HIV unexposed in Kenya (HOPE study). BMJ Open. 2024;14(6):e081975. doi: 10.1136/bmjopen-2023-081975 38844397 PMC11163661

[22] Center for Epidemiological Studies Depression (CESD) [Internet]. https://www.apa.org. [cited 2024 Aug 10]. Available from: https://www.apa.org/pi/about/publications/caregivers/practice-settings/assessment/tools/depression-scale

[23] Spitzer RL, Kroenke K, Williams JBW, Löwe B. A brief measure for assessing generalized anxiety disorder: the GAD-7. Arch Intern Med. 2006;166(10):1092–7. 16717171 10.1001/archinte.166.10.1092

[24] Kroenke K, Spitzer RL, Williams JB. The PHQ-9: validity of a brief depression severity measure. J Gen Intern Med. 2001;16(9):606–13. doi: 10.1046/j.1525-1497.2001.016009606.x 11556941 PMC1495268

[25] Short screening scales to monitor population prevalences and trends in non-specific psychological distress | Psychological Medicine | Cambridge Core [Internet]. [cited 2024 Aug 10]. Available from: https://www-cambridge-org.offcampus.lib.washington.edu/core/journals/psychological-medicine/article/short-screening-scales-to-monitor-population-prevalences-and-trends-in-nonspecific-psychological-distress/F141675CCD0E08C0FB98E01C006B4E0D 10.1017/s0033291702006074 12214795

[26] Sekhon M, Cartwright M, Francis JJ. Development of a theory-informed questionnaire to assess the acceptability of healthcare interventions. BMC Health Serv Res. 2022;22(1):279. doi: 10.1186/s12913-022-07577-3 35232455 PMC8887649

[27] Braun V, Clarke V. Successful Qualitative Research: A Practical Guide for Beginners. 2013 [cited 2025 Nov 28];1–400. Available from: https://www.torrossa.com/en/resources/an/5017629

[28] Antoniou E, Stamoulou P, Tzanoulinou M-D, Orovou E. Perinatal Mental Health; The Role and the Effect of the Partner: A Systematic Review. Healthcare (Basel). 2021;9(11):1572. doi: 10.3390/healthcare9111572 34828618 PMC8624285

[29] Shorey S, Chan V. Paternal mental health during the perinatal period: A qualitative systematic review. J Adv Nurs. 2020;76(6):1307–19. doi: 10.1111/jan.14325 32043615

[30] McCann JK, Freire S, de Oliveira CVR, Ochieng M, Jeong J. Father involvement is a protective factor for maternal mental health in Western Kenya. SSM Ment Health. 2024;5:100318. doi: 10.1016/j.ssmmh.2024.100318 38910840 PMC11192501

[31] Drysdale RE, Slemming W, Makusha T, Richter LM. Father involvement, maternal depression and child nutritional outcomes in Soweto, South Africa. Matern Child Nutr. 2021;17 Suppl 1(Suppl 1):e13177. doi: 10.1111/mcn.13177 34241955 PMC8269140

[32] Martin RCB, Brock RL. The importance of high-quality partner support for reducing stress during pregnancy and postpartum bonding impairments. Arch Womens Ment Health. 2023;26(2):201–9. doi: 10.1007/s00737-023-01299-z 36795132 PMC10716682

[33] Abas M, Bowers T, Manda E, Cooper S, Machando D, Verhey R, et al. “Opening up the mind”: problem-solving therapy delivered by female lay health workers to improve access to evidence-based care for depression and other common mental disorders through the Friendship Bench Project in Zimbabwe. Int J Ment Health Syst. 2016;10:39. doi: 10.1186/s13033-016-0071-9 27175215 PMC4865009

[34] Fernando S, Brown T, Datta K, Chidhanguro D, Tavengwa NV, Chandna J, et al. The Friendship Bench as a brief psychological intervention with peer support in rural Zimbabwean women: a mixed methods pilot evaluation. Glob Ment Health (Camb). 2021;8:e31. doi: 10.1017/gmh.2021.32 34513000 PMC8392686

[35] Hosaka KRJ, Kang E, Huff J, Shaw S, Duomai S. Livelihood intervention and mental well-being among women living with HIV in Delhi. AIDS Care. 2021;33(8):1037–43. doi: 10.1080/09540121.2020.1837336 33103920

[36] Waldron EM, Burnett-Zeigler I, Wee V, Ng YW, Koenig LJ, Pederson AB, et al. Mental Health in Women Living With HIV: The Unique and Unmet Needs. J Int Assoc Provid AIDS Care [Internet]. 2021 Jan 21 [cited 2024 July 18];20:2325958220985665. Available from: https://www.ncbi.nlm.nih.gov/pmc/articles/PMC7829520/10.1177/2325958220985665 PMC7829520 33472517

[37] Yousuf A, Musa R, Isa MLM, Arifin SRM. Anxiety and Depression Among Women Living with HIV: Prevalence and Correlations. Clin Pract Epidemiol Ment Health. 2020;16:59–66. doi: 10.2174/1745017902016010059 32742296 PMC7372730

