# Correction: “When a man is stressed, it replicates in the house”: Kenyan women’s perspectives on the influence of male partners on perinatal mental health among women affected by HIV

**DOI:** 10.1371/journal.pgph.0006451

**Published:** 2026-05-08

**Authors:** Hellen Moraa, Joan Mutahi, Winnie Atieno, Grace John-Stewart, John Kinuthia, Manasi Kumar, Mary Marwa, Ben Odhiambo, Felix Abuna, Jillian Pintye, Dalton Wamalwa, Gabrielle O’Malley, Keshet Ronen, Irene Njuguna, Anna Larsen

In the Funding statement, the grant number from the funder National Institute of Health (US) is listed incorrectly. The correct grant number is: P30MH123248.
