# Supplementary material for: “When a man is stressed, it replicates in the house”: Kenyan women’s perspectives on the influence of male partners on perinatal mental health among women affected by HIV
Source: PLOS Glob Public Health. 2026 Mar 4;6(3):e0006047. doi: 10.1371/journal.pgph.0006047 (PMC12959676; doi:10.1371/journal.pgph.0006047)
Supplement: S1 Table — (DOCX) [file pgph.0006047.s001.docx]

**S1 Table. Additional quotations depicting key themes from qualitative analysis**

| **Themes and Illustrative Quotations** |
| --- |
| **Theme: Drivers and symptoms of mental distress** |
| ***“****Because she could see I was down, at times I was just bored; I didn’t have a big issue but I was just bored. She would ask if I was okay and I would say I was okay from the surface but deep down I felt like even if someone sees me they would know I’m not okay****.* (IDI participant #7, WNLHIV*)*** |
| **“***You just feel like your is head full, you don’t even know what to do because your mind is not working well.****”* (IDI participant #16, WNLHIV)** |
| **“***When you are pregnant and stressed, those are so many burdens. You will lack appetite, lose weight and even end up lacking blood*.”  **(IDI participant #9, WLHIV*)*** |
| ***“****Somebody may be having stress, and you may never know that you are stressed, or something is distracting your mind until the moment … you are taking something; you feel like there is loss of appetite****.”* (IDI participant #19, WLHIV)** |
| **Theme: Sources of mental distress** |
| **“***When one is pregnant, they sometimes act crazy. You may wake up and just blow up for no good reason. So, the counselling really helps****.”* (IDI participant #5, WNLHIV)** |
| **“***It is usually hard, I have passed through hard times but I thank God, at times I pray but there are times I am not able to pray, those times I leave for God to do as He pleases. I have children and my husband died, though he never told me anything before he died. While he was alive and I was pregnant with the child that he left me with when he was still a baby, I went to hospital and it is when I found out that I had HIV. When I told him, he wasn’t surprised and he never said anything nor did he change. What he told me is that those doctors are lying and maybe because I had some problems back at home, it could have been the reason my blood was found to be that way. I was stupid and just took it that way, we continued staying and I don’t know what he thought, I had trusted him and never thought he could be unfaithful, so I just stayed*. **(FGD 1, participant 1, WLHIV)** |
| **Subtheme: Tension related to HIV disclosure** |
| **“***I became open and told her because when we used to stay with my husband… he was HIV Positive. Now this thing was really disturbing us inside our house- one person is positive while the other is not. It would be quarrels that led to chaos but God came through for me until (name of nurse) taught me until she enrolled me into drugs and told me that, “There is no day that you will be sick and I want you to be free of stress*” **(FGD 3 participant #2, WNLHIV)** |
| **Theme: Men frequently mentioned as a source of mental stress** |
| **Sub-theme: Abandonment** |
| **“***Many men cheat when the woman is pregnant and this hurts a lot and may lead to stress and depression. When you are pregnant you can't think of revenging and just want to find a way of raising your children. I got to a point I was just talking to myself because it is not easy to share some of these things because some judge you****”* (FGD 2 participant #9, WNLHIV)”** |
| **“***Now it came to a point where we had a disagreement with my husband and he abandoned me with the baby and moved to Sindo, where he was working. Now I started developing stress, I could not sleep I am just awake, I cannot even chat, I was stressed-out and went back to the hospital****.*” (IDI participant # 13, WNLHIV)** |
| **“***I was married, and I stayed in that marriage for seven years where the Lord blessed me with a daughter. On the seventh year, my husband started changing a bit, that was during the time we had COVID. At times he would tell me that he was caught up in traffic or with the curfew and had gotten somewhere to sleep. ….. In January 2021, he picked up his stuff and left. When he left, I didn’t even know that I was pregnant with the son I now have … I tested and found out I was pregnant. I thought of killing myself and threw myself into a well, but God helped us, and we were rescued alive****”* (FGD 1, participant #5, WLHIV)** |
| **Sub-theme: Lack of financial support** |
| **“***It is true that as mothers we go through several challenges and especially when pregnant or postpartum. Today’s men want working women, so for me I think even if you have a stubborn pregnancy, try do something that can earn you something because if you don’t this man sees you as a burden … I urge women to have something to do to earn an income and be wise to save. When I was pregnant with the child I came with, I was stressed because he packed things and went to the village and told me to join him there. When I thought about going there, I knew that it would be a life of struggle. I went and looked for a job and up to now I am still doing it, I asked God to help me be able to feed and do everything for my children through that job, I worked until the last day (of pregnancy****)*. (FGD 2 participant #4, WNLHIV)** |
| **Sub-theme: Verbal or physical abuse** |
| **“***There are other times I could feel my heart beating. I feel I am not okay. So, even when my husband would come to the house quarreling, after I over think, I would be in shock and start trembling****.” (*IDI participant #7, WNLHIV)** |
| **Theme: Men too are hurting** |
| *" Men mostly like keeping quiet with their issues. An issue can bother him, but he just keeps it to himself. You may find that he cannot talk about the issue bothering him and instead it hurts you. It is you whom he later comes to release the stress to. There are some who keep silent without talking as others like chaos that even if it is a mere cup that he finds on the table... it becomes an issue. When food delays, which is his fault coming late with food, when it’s not ready on time he leaves it for you. Such kind of things. Now how they handle issues differs with how they are. But mostly it is us who get hurt instead***. (FGD 3 participant #1, WNLHIV)** |
| ***“****If one also has an understanding husband, this can also make them very happy during pregnancy, the husband doesn't contribute to her stress***.” (FGD1 participant #1, WLHIV)** |
| **Theme: Coping strategies** |
| “*I think it is better to share your stress with someone because if you keep it in, it shall harm you. When you share with people, they give you different advice and after a while you become okay***.” (FGD 2 participant #2, WNLHIV)** |
| **“***as women, when we are stressed, we usually share with our close friends. You could be thinking your issue is the hardest but as you share and they tell you what they are going, you come to see that yours is even better*” **(FGD 2 participant #9, WNLHIV)** |
| **“***I was so stressed and kept to myself because I felt no one cared. I got to a place and would just go and spend my time in church, the pastor would talk to me and when I would go back home, I got to know how to control myself*.” **(FGD 2 participant #9, WNLHIV)** |
| ***“****I had even decided to die and get out of earth. Now the way she [the counsellor] talked to me made my thoughts move from doing something bad in my life***.” (IDI participant #13, WNLHIV)** |
